# Assessment of drug permeability using a small airway microphysiological system

**DOI:** 10.3389/fphar.2025.1621775

**Published:** 2025-07-17

**Authors:** Robert M. Geiger, Shekh M. Rahman, Md Shadiqur Rashid Roni, Catherine Sullenberger, Sabyasachy Mistry, Katherine Shea, Isra Tariq, Omnia A. Ismaiel, Murali K. Matta, Paula L. Hyland, Sasha Berdichevski, Alexandre J. S. Ribeiro, Ksenia Blinova, Wenlei Jiang, Ross L. Walenga, Bryan Newman, Donna A. Volpe, Kevin A. Ford

**Affiliations:** ^1^ Division of Applied Regulatory Science, Office of Clinical Pharmacology, Office of Translational Sciences, Center for Drug Evaluation and Research, United States Food and Drug Administration, Silver Spring, MD, United States; ^2^ Emulate, Inc., Boston, MA, United States; ^3^ SciBer Ltd., Cambridge, United Kingdom; ^4^ Office of Research and Standards, Office of Generic Drugs, Center for Drug Evaluation and Research, United States Food and Drug Administration, Silver Spring, MD, United States

**Keywords:** microphysiological system, permeability, regulatory science, small airway, inhaled drugs

## Abstract

**Background:**

There is a need to reliably predict the permeability of inhaled compounds during the development of new and generic drugs. A small airway microphysiological system (MPS) that can recapitulate the pulmonary air-liquid interface (ALI) with primary epithelial and vascular endothelial cell layers may provide a more physiologically relevant environment for measuring drug permeability than simpler two-dimensional *in vitro* cell culture platforms. Therefore, we evaluated the use of a small airway MPS to measure the permeability of inhaled drugs.

**Methodology:**

Primary human lung epithelial cells were seeded onto the top channel of the chip and cultured for 14 days at ALI to promote monolayer differentiation, followed by addition of endothelial cells into the bottom channel. Due to the non-specific binding properties of polydimethylsiloxane (PDMS), a drug absorption study was conducted to quantify non-specific binding to the material. Drug permeability was evaluated by passing each compound (10 µM) through the top channel and measuring the amount of drug that permeated into the bottom channel over the time course of 30, 60, 120, and 180 min.

**Results:**

Confocal micrographs demonstrated the presence of tight junctions along with basal, goblet, and ciliated cells in the top channel and attachment of endothelial cells in the bottom channel. Insignificant nonspecific binding to the MPS was observed with albuterol sulfate, formoterol fumarate, and olodaterol hydrochloride (HCl), while fluticasone furoate showed significant nonspecific binding as only 6%–44% of the drug was recovered at 30 and 120 min, respectively. As a result, fluticasone furoate was excluded from further analysis. Permeability studies estimated an apparent permeability (P_app_) of 1.02 × 10^−6^ cm/s for albuterol sulfate, 0.0813 × 10^−6^ cm/s for olodaterol HCl, and 2.44 × 10^−6^ cm/s for formoterol fumarate.

**Discussion:**

Taken together, the small airway MPS recapitulated relevant cell types and many morphological features in the lung. The apparent permeabilities measured indicated that albuterol sulfate and formoterol fumarate would be categorized as highly permeable, while olodaterol HCl would be categorized as a low permeable drug.

## Introduction

Microphysiological systems (MPS) or organs-on-chips have been identified as potential alternatives to traditional *in vitro* and *ex vivo* models for preclinical drug development (Ingber, 2022). MPS recapitulate “functional features of a specific tissue or organ of human or animal origin by exposing cells to a microenvironment that mimics the physiological aspects important for their function or pathophysiological condition” as defined by the U.S. Food and Drug Administration (FDA) (National Academies of Sciences and Medicine, 2021). Furthermore, MPS improve on conventional *in vitro* models for drug permeability studies by more accurately recapitulating key aspects of human physiology (via incorporation of 3D tissue architecture, dynamic fluid flow, and the use of relevant human cells) (Ingber, 2022; Nawroth et al., 2023; Wang et al., 2020). Given their enhanced physiological relevance, MPS may have a role as a drug development tool in preclinical pharmacokinetic studies.

Drug permeability is an important biopharmaceutical property to consider when developing inhaled drugs because it influences absorption and bioavailability. While there are *in vitro* models available to evaluate this parameter, there are few physiologically relevant models for lung tissue. For orally ingested compounds, the well-known intestinal Caco-2 membrane insert model is used to measure the apparent permeability (P_app_), the rate a drug crosses a biological barrier in an *in vitro* model. (Larregieu and Benet, 2013; 2014). However, this model fails to recapitulate relevant physiological features found in the lung, such as the air-liquid interface (ALI), the presence of mucin, or relevant cell types such as goblet, ciliated, or basal cells and therefore would be inappropriate for evaluating the permeability of inhaled drugs. Other membrane insert models use lung epithelial cell lines like Calu-3 or 16HBE14o cells to recapitulate the barrier between the air and vasculature (Cingolani et al., 2019; Dutton et al., 2020; Ehrhardt et al., 2005). However, these models are monolayers and do not include endothelial cells. More physiologically relevant membrane insert models use primary human epithelial and microvascular endothelial cells from the lung; however, they do not apply mechanical cues like stretch and fluid flow to the cells, which was shown to improve differentiation (Nawroth et al., 2023; Rahman et al., 2025). Other models used to evaluate drug permeability in the lung include the *ex vivo* isolated perfused rat lung model; however, this model only lasts a few hours in culture and involves animal use (Ainslie et al., 2019; Akhtar, 2015; Eriksson et al., 2018; Hiemstra et al., 2018; Selo et al., 2021). Furthermore, there are many species-specific differences between the respiratory systems of animal models and humans to consider. For example, rodents often have different architecture and cellular composition compared to humans (Frohlich, 2024). Additionally, rodents have fewer bronchioles resulting in lower drug deposition compared to humans (Frohlich, 2024). To address these potential limitations, new human *in vitro* models are needed to better evaluate the permeability of inhaled drugs.

In this study, a novel, small airway MPS was evaluated to measure the apparent permeability (P_app_) of inhaled drugs. This microfluidic system contains two adjacent channels separated by a porous membrane within microfluidic circuits composed of polydimethylsiloxane (PDMS) where lung epithelial cells are seeded into the apical (top) channel and lung microvascular endothelial cells are seeded in the basolateral (bottom) channel (Benam et al., 2016). The MPS maintains the small airway epithelial cells at ALI to generate the relevant cell types found in the lung including goblet, ciliated, and basal cells. Unlike the *ex vivo* rat lung model, this MPS uses primary human cells obtained from the lung or microvasculature, thus eliminating any species-specific differences. In addition, others have successfully used a similar gut MPS to evaluate the permeability of orally ingested drugs, suggesting that inhaled drug permeability can be evaluated using the small airway MPS (Zhang et al., 2024). This approach is also consistent with the FDA’s goal of reducing, refining, and replacing animal models (3Rs) and identifying new methodologies to help evaluate the safety and efficacy of new drugs (Avila et al., 2020; Avila et al., 2023; Wange et al., 2021). Taken together, the small airway MPS may be an alternative model for measuring the permeability of inhaled drugs.

MPS have the potential to be used as drug development tools; however, there is little guidance currently available to assist in the determination of quality, composition, and robustness of these platforms (Fowler et al., 2020). Therefore, our characterization was aimed to identify quality control attributes to consider for evaluating the apparent permeability of inhaled drugs. For example, the cellular composition was evaluated to ensure the cell types are observed in the MPS and an intact cell barrier is formed prior to performing the permeability study. Additionally, since the chip is made of PDMS, a silicone polymer well-known to bind many lipophilic drugs, and adsorb proteins, we also evaluated drug absorbance to identify any drug loss due to non-specific binding to the MPS (Chumbimuni-Torres et al., 2011; Leung et al., 2022; van Meer et al., 2017). Collectively, it is anticipated that these quality control attributes may be useful to drug developers and regulators.

To determine if the small airway MPS may be used for measuring drug permeability, we evaluated the *in vitro* permeability of several inhaled drugs used in drug products to treat symptoms of chronic obstructive pulmonary disease (COPD) and asthma, namely, albuterol sulfate, fluticasone furoate, formoterol fumarate, and olodaterol HCl. They have been widely studied in clinics and in *in vitro* studies (Cingolani et al., 2019; Ehrhardt et al., 2005; Eriksson et al., 2018; Patel et al., 2016). Using the apparent permeability results, the drugs were categorized using the recently proposed inhaled biopharmaceutical classification system (iBCS) and compared to an *ex vivo* isolated perfused lung (IPL) rat model from another study (Eriksson et al., 2018; Hastedt et al., 2022). Here, we evaluated a small airway MPS that recapitulates the human ALI barrier to assess inhaled drug permeability, addressing key gaps in preclinical models.

## Materials and methods

### Small airway MPS culture methods

**FIGURE 1:**
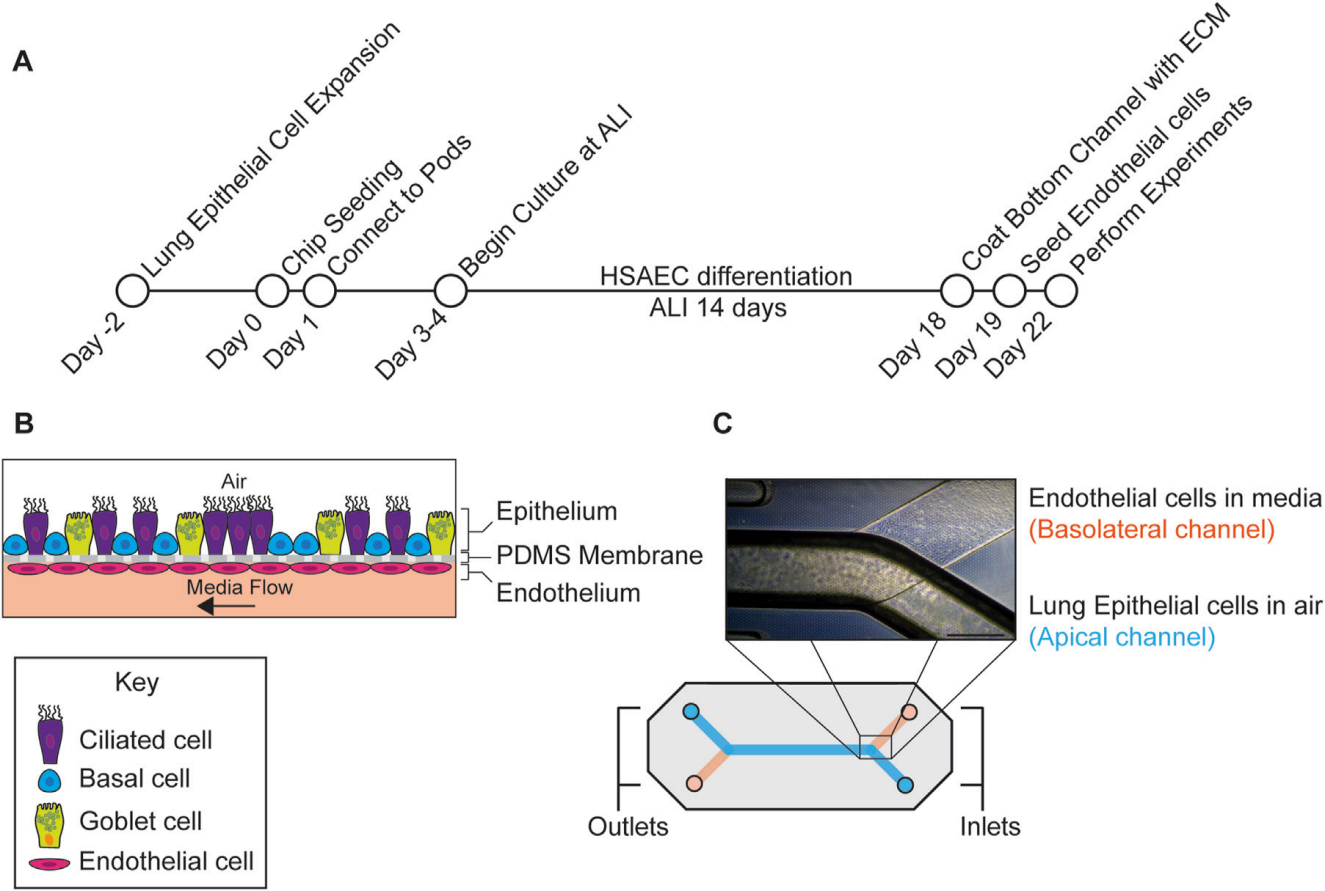
Outline of experimental workflow. **(A)** Protocol used to generate the small airway MPS. The MPS was seeded with human small airway epithelial cells (HSAECs) in the apical channel and differentiated for 14 days at the air-liquid interface (ALI). After differentiation, microvascular endothelial cells were added in the basolateral channel followed by drug permeability studies on day 22 post seeding. **(B)** Diagram depicting lung cell types in respective channels. **(C)** Diagram of the chip along with a phase contrast image of the assembled chip with epithelial cells in the top channel and endothelial cells in the bottom. Scale Bar = 500 µm.

The experimental protocol is shown in Figure 1A. Primary human small airway epithelial cells (Cat# FC-0016, Lot # 09218, 09,438) were purchased from (Lifeline Cell Technology, Frederick, MD). Small airway epithelial cells were sourced from donor: 09438 (Asian male, 48 years old) and donor: 09218 (Hispanic male, 35 years old). Cells from donors 09218 and 09438 were used for drug permeability, barrier integrity and microscopy studies, cells from donor 09438 were used for mucin analysis, H/E staining and gene expression studies. Both donors were sourced from the same supplier and extracted from the same region of the lung. Furthermore, both donors were observed to generate a confluent monolayer of cells while maintaining an air-liquid interface. The primary cells were thawed into a T75 flask coated with Collagen IV (Sigma, St. Louis, MO) (0.15 mg/mL), grown in small airway growth media (Promocell GmbH, Heidelberg, Germany), fed the day after thawing and then every other day until 80%–90% confluency. One day before seeding with cells, S1 chips (Emulate, Boston, MA) were activated with 1 mg/mL ER1, (a required proprietary surface activation reagent, Emulate), in both the apical (top) and basolateral (bottom) channels and coated with 0.5 mg/mL Collagen IV (Sigma) in Dulbecco’s phosphate buffered saline (dPBS, Corning, NY) in the apical channel, the basolateral channel kept empty. Cells were then detached using Tryple E™ (Thermo Fisher Scientific, Waltham, MA), and 35 μL of cells were seeded into the apical channel of the chip at a concentration of 3 × 10^6^ cells/mL with PneumaCult™ ALI complete media (STEMCELL™ Technologies, Cambridge, MA) supplemented with a photostable synthetic stable retinoid (EC23) (50 nM) (Tocris), epidermal growth factor (EGF) (10 ng/mL) (Promocell), hydrocortisone (0.48 μg/mL) (STEMCELL™ Technologies) and heparin (4 μg/mL) (STEMCELL™ Technologies). Media was prewarmed for 1 h at 37°C and degassed with Steriflip 50 mL filters (Sigma). Following incubation overnight at 37°C, chips were gently washed with 200 µL PneumaCult™ ALI complete media and drops of media were placed on each of the inlet and outlet ports of the chip. Pods (device used to contain the media) (Emulate) were primed twice with PneumaCult™ ALI media supplemented with EC23 and EGF using the Emulate Zoë apparatus. Chips were connected to the pods and a regulate cycle (setting used to help remove any air bubbles in the fluidics) was performed. 48 h after seeding, a second regulate cycle was completed. After the chips were 100% confluent, an ALI was established by removing the media from the top reservoir inlet and outlet and running the flow at 600 μL/h for 5 min to purge the remaining media in the channel and fluidics. A liquid plug using PneumaCult™ media was created by adding 1 mL to the inlet and outlet reservoir of the top channel. In the initial development of the protocol, chips were maintained at ALI for 14–18 days and washed every 2–4 days, however after optimization, chips were maintained for 14 days at ALI (Figure 1B). While some studies have indicated that dynamically stretching the cells may improve differentiation (Nawroth et al., 2023), this may not be required as others have successfully cultured the small airway chip without applying stretch to the cells (Benam et al., 2016). In light of this, we did not apply this mechanical stress to the cells in our protocol.

**FIGURE 2:**
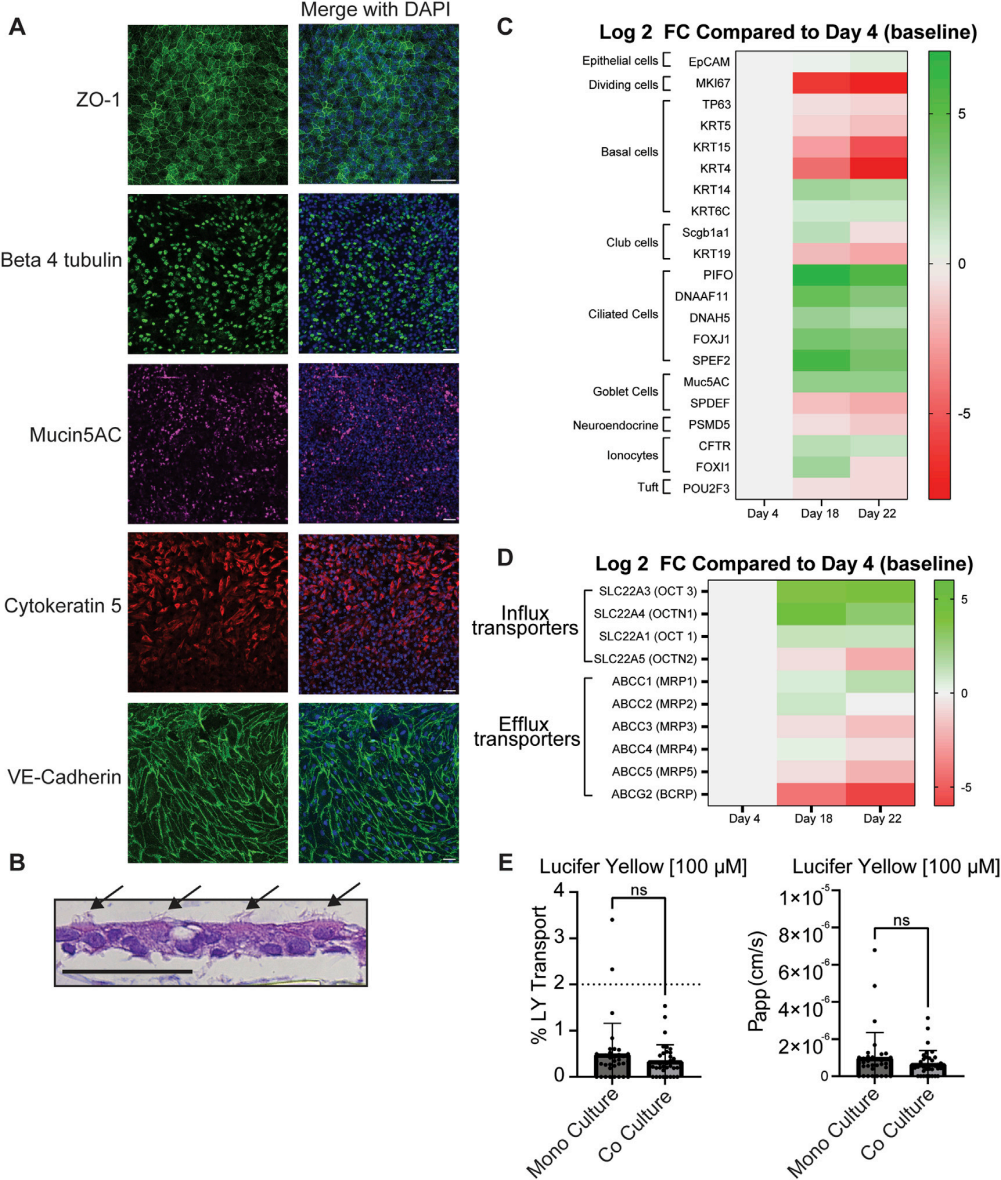
Characterization of small airway MPS. **(A)** Representative images of markers for tight junctions (ZO-1), ciliated cells (beta 4 tubulin)), basal cells (cytokeratin 5), goblet cells (Mucin5AC) and endothelial cell adherens junctions (VE-cadherin). All antibodies were diluted to (1:100) n = 9–11. **(B)** Further evidence of ciliated cells identified by arrows are shown by H/E stain **(B)** n = 3 chips. **(C)** Heat map of differential expression of cell type, and proliferation markers and **(D)** drug transporters. Heat map shows the average log2 fold change of transcript levels on Day 18 and Day 22 compared to Day 4 (Day 0 of ALI). Green and red indicate upregulation and downregulation respectively. Only targets that were detected below the Ct cuff-off (35) in 50% of arrays were used to calculate fold changes. A Student’s t-test was used to determine statistical significance (See Supplementary Tables S2-S3). A single differentiation was performed with RNA collected from three chips on each day and transcript levels were evaluated in three independent RT-qPCR reactions from each chip. **(E)** The integrity of the cell barrier in the small airway MPS was probed with lucifer yellow (LY) at 100 µM and the apparent permeability (P_app_) was calculated based on Equation 1. Samples that had a concentration below the limit of quantitation were set to 0.0 Data represent the mean ± standard deviation (SD); n = 35 biological replicates (100 µM); unpaired t-test, ns = no statistical difference. Scale Bar = 50 µm.

Lung microvascular endothelial cells (Lonza, Cat# CC-2527, Cambridge, MA, Lot# 21TL138967, 22TL024424, 23TL003044, 23TL206653) were thawed into a T75 flask and fed every other day until 80%–90% confluency. After 14 days of differentiation, chips were detached from the pods, the top channel was flooded with PneumaCult™ ALI media and endothelial extracellular matrix (ECM) ((Collagen I (100 μg/mL) (Sigma), fibronectin (50 μg/mL) (Sigma), and laminin 50 μg/mL (Sigma)) was added to the basolateral channel and incubated overnight at 37°C. Endothelial cells were added to the chip after small airway epithelial cells as they did not require differentiation. Seeding both cell types at different timepoints likely had minimal impact on barrier function or cross talk as an intact barrier was observed before and after endothelial cells were added (Figure 2E). After growing endothelial cells in a T75 flask for 4 days, cells were removed with Tryple E™ (Thermo Fisher) and 20 µL of (5 × 10^6^ cells/mL) were seeded into the basolateral channel of the chip in endothelial cell media (Lonza). After seeding, the chip was immediately inverted for the cells to attach to the top of the basolateral channel and then incubated for 5 h at 37°C. After incubation, chips were re-inverted and gently washed with endothelial cell media in the basolateral channel and PneumaCult™ ALI media in the top channel. The following day, the chips were washed and reconnected to the pods. After another regulate cycle was performed, ALI was reestablished using a 50:50 mix of PneumaCult™ ALI media and endothelial cell media in the bottom channel with a flow rate of 30 μL/h. A phase contrast image of the chip with both epithelial cells and endothelial cells is shown in (Figure 1C).

### Microscopy

The chips on day 22–28 were washed with dPBS (Corning) and fixed using 4% paraformaldehyde (PFA) (Thermo Fisher) for 20 min. Chips were permeabilized for 30 min with 0.1% Triton X-100 (Sigma) and blocked with 10% goat serum overnight at 4°C. Chips were then cut in half followed by incubation with primary antibody at (1:100), v/v ratio in 1% bovine serum albumin (BSA), w/v (Sigma) at 4°C overnight. Antibodies used in this study were beta 4 tubulin (Cat# ab11315, Abcam), ZO-1 (Cat# 33–9,100, Invitrogen), MUC5AC (Cat# AB3649, Abcam), cytokeratin 5 (Cat# ab52635, Abcam) and VE-Cadherin (Cat# 14–1,449-82, Invitrogen) Chips were washed with dPBS and incubated with secondary antibody at (1:500), v/v ratio for 2 h and a DAPI stain to identify cell nuclei. Chips were kept in dPBS until examined under a confocal laser scanning microscope (Carl Zeiss Microscopy, LLC., White Plains, NY).

### Hematoxylin/eosin (H/E) staining

Chips were fixed with 4% paraformaldehyde, cut into 4 µm-thick paraffin sections, mounted on glass slides, and dried overnight at room temperature. Sections were stained with H/E using a routine automated staining protocol on a Leica ST5020 slide Stainer with Leica SelecTech (Deer Park, IL) staining reagents. Coverslips were placed on stained slides with permanent mounting medium.

### Mucin quantitation

Mucin was allowed to accumulate in the apical channel for the last 3 days of ALI (days 16–18). Previous studies have observed mucin after 14 days of culturing primary lung epithelial cells at ALI using this MPS (Plebani et al., 2021). Mucin was harvested by disconnecting the chip from the pod, followed by addition of dPBS (Corning) to the apical channel and incubating for 1 h. The mucin wash was then removed and stored at −30°C. Mucin was quantitated using an Alcian blue (Vector Laboratories, Newark, CA) colorimetric absorbance assay. In brief, standards were made from bovine mucin (Sigma) and incubated with alcian blue alongside samples. Samples and standards were washed 3× with PBS to help remove debris that was not mucin. Following the final wash, samples were resuspended in 90 µL of 10% sodium dodecyl sulfate (SDS). 90 μL of samples and standards were placed in a 96 well plate (Thermo Scientific). Absorbance was read at 620 nM using the Tecan Spark 10M plate reader (Woburn, MA). The amount of Alcian blue measured was normalized to the 30 uL volume collected from the wash (Rahman et al., 2025).

### Small airway MPS focused gene expression profiling

Total RNA from epithelial cells was extracted from chips on Days 4, 18, and 22 of cell culture using the Pure Link RNA mini kit (Thermo Fisher) and quality controlled as detailed in Supplementary Section S1–S4. Total RNA was converted into template cDNA (Qiagen QuantiNova reverse transcription kit) and then characterized using a custom qPCR Array (Qiagen) and the QuantiNova SYBR Green qPCR Master Mix on the QuantStudio 12K Flex PCR System (Applied Biosystems™, Waltham, MA) according to manufacturer’s instructions. The custom PCR array was designed using the QuantiNova LNA PCR Array System (Qiagen LLC, Germantown, MD) and included 84 genes of interest to screen relative gene expression levels of transporter genes of interest (*e.g*., *SLC22A1, SLC22A3, SLC22A4*, *SLC22A5*) as well as cell-type specific genes (*e.g*., *EpCAM*), and proliferation (*e.g*., *MKI67*) and differentiation (*e.g*., *TP63*) gene markers (Supplementary Table ST1). The contents of the custom PCR Array, including five housekeeping genes (HKGs) and three controls, are described in detail in Supplementary Table ST1. RT-qPCR was performed at least in triplicate for each RNA sample and data analysis was performed using GeneGlobe (Qiagen) (https://geneglobe.qiagen.com/re). A cycle threshold (Ct) of 35 was set as the cut-off value for gene expression (Qiagen, 2015; Rubiano et al., 2021). Genes, with Ct values >35 cycles were considered non-detectable. Data were normalized to the most stable HKGs across the experimental conditions and differential gene expression between Days 18 or 22 vs Day 4 was determined (Supplementary Section S3A). Fold changes were only calculated if the target was detected (Ct < 35) in at least 50% of replicas/PCR arrays for both days. Statistical significance was determined using a Student’s t-test and a Bonferroni-corrected threshold for multiple testing (i.e., 0.05/67 expressed targets). T-test *p-values* < 7.46E-04 were considered significant.

### Barrier integrity study

Barrier integrity was assessed with Lucifer yellow (LY) (Thermo Fisher) diluted in PneumaCult™ basal media (STEMCELL™ Technologies) without any supplements. LY 10 µM or 100 µM was passed through the apical channel and PneumaCult™ basal media at 120 μL/h for 2 h as recommended by Emulate. Samples were collected in a clear bottom 96 well plate (Thermo Fisher) and fluorescence was quantified in a spectrometer (Tecan Spark 10M) at 428 nm excitation, 536 nm emission using a standard curve.

### Evaluation of drug loss due to absorption (non-specific binding) to MPS chip

Drug was diluted in PneumaCult™ basal media without supplements to help avoid drug protein binding. Stock solutions of drugs were made the day of the study or the day before and frozen overnight at −30°C. One day prior to performing the absorbance study, chips from the compound distribution kit (CDK) with no cells (Emulate, Boston, MA) were connected to pods and primed with PneumaCult™ basal media. Overnight, a regulate cycle was performed. The following day, drug diluted in PneumaCult™ media was flowed at 250 μL/h into both the top and bottom channel for 180 min. Samples were harvested under low lighting every 30, 60, 120, 180 min in LoBind tubes (Eppendorf, Enfield, CT), flash frozen and stored at −80°C. Samples were quantitated with liquid chromatography tandem mass spectrometry (LC-MS/MS).

### Drug permeability study

Drug was diluted to a stock concentration with sterile distilled water or dimethyl sulfoxide (DMSO) (Sigma). The stock was diluted to 10 µM using PneumaCult™ basal media (STEMCELL™ Technologies) with no supplements added. The pod and chip were primed with the drug at 600 μL/h for 5 min. The flow-through was then discarded from the outlet reservoirs. Following priming, the flow rate was set to 250 μL/h in both channels and, 50 µL samples were collected at 0, 30, 60, 120 and 180 min timepoints. All samples were collected in LoBind tubes (Eppendorf), flash frozen on dry ice and immediately stored at −80°C. Drug concentrations in the samples were quantitated using LC-MS/MS.

### Data analysis

The apparent permeability (P_app_) for both the drug permeability and tracer assays was calculated using Equation 1, where Q_R_ and Q_D_ are the flow rates in the receiving and dowsing channel respectively, C_R,0_ and C_D,0_ are the drug concentrations recovered from both the receiving and dosing channel respectively and SA is the surface area of the chip (*i.e*., 0.171 cm^2^) (Zhang et al., 2024).

$$P_{app}=-\frac{Q_{R}\;*\;Q_{D}}{SA\;*\;\left(Q_{R}+Q_{D}\right)}*\ln\left[1-\frac{C_{R,0}\;*\;\left(Q_{R}+Q_{D}\right)}{\left(Q_{R}\;*\;C_{R,0}+Q_{D}\;*\;C_{D,0}\right)}\right]$$ (1)

The cumulative amount of drug transport was calculated using Equation 2 adapted from (Salminen et al., 2023). Where.

• n is the number of timepoints (1, 2, 3, 4) from each chip corresponding to 30, 60, 120, 180 min.
• M_i_ is the number of moles in the sample,
• V_s_ is the sample volume (50 μL)
• V_i_ is the total amount of drug or media that has flowed during a specific timepoint, given our flow rate of 250 μL/h, either 125 μL for a 30 min interval or 250 for a 60 min interval.

The Dose number was calculated using Equation 3 (Hastedt et al., 2024). Where.

• M_r_ is the regional dose.
• V_f_ is the volume of fluid available for dissolution.
• C_si_ is the solubility of the drug.

$$Cummulative\;Amount=\sum_{i=1}^{n}\left(\frac{M_{i}}{V_{s}}\right)V_{i}$$ (2)

$$D_{O_{i}}=\frac{M_{r}/V_{f}}{C_{s_{i}}}$$ (3)

### LC-MS/MS analysis

LC-MS/MS methods were developed and validated to measure the tested compounds in the study media. Linearity was established over concentration ranges of 10.0–2000.0 nM, 50.0–2000.0 nM, 7.81–2000.0, and 7.81–2000.0 nM for albuterol sulfate, fluticasone furoate, formoterol fumarate, and olodaterol HCl, respectively. Calibration standards (CSs), quality control (QCs) samples and study samples in PneumaCult™ basal media were prepared by dilution with a combination of water and organic solvents. 3-fold volumes of 5% BSA was added to formoterol fumarate samples and 2-fold 5% BSA was added to olodaterol HCl samples to improve drug stability while being prepared for analysis. A volume of 0.5–1.0 µL was injected into LC-MS/MS.

### Statistical analysis

Statistical analysis was performed using GraphPad Prism (version 10.4, Boston, MA) as described in the figure legends. Outliers were identified using a Rout outlier test. Images were analyzed in the open-source image processing software package Fiji. Diagrams of chips were made in Adobe Illustrator^®^ (Version 28.5, San Jose, CA).

## Results

The goal of this study was to evaluate the potential of the small airway MPS for measuring the permeability of inhaled drugs and to characterize the MPS to identify quality control criteria. The small airway MPS was first evaluated for relevant cell types and features that are observed *in vivo* including basal, goblet, ciliated cells, tight junctions, and mucin. Next, since the chips were used for drug permeability studies, we measured the integrity of the cell bilayer using Lucifer yellow (LY) to ensure barrier integrity. Chips with low LY permeability were used in the drug permeability experiments. We then compared our results to the *ex vivo* rat isolated perfused lung model published for the same application.

### Characterization of the small airway MPS

The small airway MPS was characterized to ensure that physiologically relevant cell types and features were present using confocal microscopy after labeling specific markers of the small airway epithelium. Immunofluorescence showed evidence of the expected cell types, including goblet (Mucin5AC), basal (cytokeratin 5) and ciliated cells (beta 4 tubulin) (Figure 2A). In order to ensure tight junction integrity, expression of zonula occludin 1 (ZO-1), a marker of tight-junctions, was observed between the epithelial cells. Additionally, expression of VE-cadherin, a marker for adherens junctions, localized between endothelial cells in the basolateral channel. Collectively, these findings demonstrate the presence of anticipated cell types in each channel and with *in vivo* like cell-cell contact. Further evidence of ciliated cells is shown using an H/E stain (Figure 2B). Mucin was also observed at a concentration of 1.12 mg/mL in the apical channel after accumulating for the last 3 days at ALI supporting the presence of functional goblet cells.

To further explore other cell types generated in the small airway MPS, we used a custom qPCR array (Supplementary Table ST1) to compare gene expression changes of functional and lung-specific cell type markers before ALI (Day 4) to immediately after ALI (Day 18), and to the day of the permeability study (Day 22). As the epithelial monolayers differentiated, the levels of basal progenitor markers like *KRT4* and *KRT15* were significantly (*t*-test p-value <7.46E-04) and strongly downregulated, while *TP63*, a pan-basal cell marker was shown to decrease (albeit only at > 50% reduction) suggesting a reduction in basal cells, consistent with their differentiation into other cell types (Figure 2C; Supplementary Tables ST2, T3). Cell type specific (CTS) gene markers for neuroendocrine (NE) cells, ionocytes, and Tuft cells were detected and differentially expressed suggesting potential changes in the presence of these cell types in the small airway MPS (Figure 2C; Supplementary Tables ST2, ST3). Evidence for club cells was also observed as the club cell marker *SCGB1A1* was significantly upregulated (FC = 2.95) on Day 18 compared to Day 4 (*t*-test, p-value = 4.33E-04) (Figure 2C; Supplementary Table ST3). Expression of *MUC5AC,* a goblet cell marker, was significantly increased on Day 18 and maintained (at nominal statistical significance) to Day 22 compared to Day 4 consistent with presence of goblet cells. Lastly, the expression of several markers for ciliated cells were observed to be upregulated on Day 18 and Day 22 compared to Day 4 (Figure 2C; Supplementary Tables ST2, ST3).

Next, the expression of the organic cation transporters (OCTs) was examined. These transporters are suggested to be involved in the transport of many inhaled drugs in the lung including those evaluated in this study (Ehrhardt et al., 2017; Ehrhardt et al., 2005; Nickel et al., 2016; Salomon et al., 2015). Genes *SLC22A1* (OCT1)*, SLC22A3* (OCT3)*, SLC22A4* (OCTN1)*, SLC22A5* (OCTN2) were shown to be expressed in lung epithelial cells from our donor; however, some were upregulated while others were downregulated during differentiation (Figure 2D; Supplementary Tables ST2, ST3). For example, *SLC22A3* (OCT3) and *SLC22A4* (OCTN1) were strongly upregulated with nominal p-value significance after ALI on both days compared to Day 4. *SLC22A1* (OCT1) also showed a trend but its upregulation (FC ≥ 2) was nominally non-significant following differentiation. In contrast, *SLC22A5* (OCTN2)*,* while still expressed (Supplementary Tables ST2, ST3)*,* showed reduced expression relative to Day 4. Similarly, drug efflux transporters *ABCC1-5* (MRP1-5) and *ABCG2* (BCRP) were also confirmed to be expressed in small airway epithelial cells but showed reduced expression relative to Day 4. Taken together, many of the same OCTs expressed *in vivo* are also expressed in the small airway MPS.

### Barrier integrity assessment

To ensure an intact barrier of cells between chips, the confluency of the cell layer between the apical and basolateral channels was probed prior to drug permeability studies using Lucifer Yellow (LY). This fluorescent dye, commonly used in permeability models, permeates areas with less cells more easily resulting in a higher P_app_ and is suggestive of a less intact barrier (Cingolani et al., 2019; Mukherjee et al., 2017). LY was added at either 100 µM (Figure 2E) or 10 µM (data not shown, as most samples using 10 µM LY were below limit of quantitation in the bottom channel). In order to obtain reliable quantitation while remaining non-toxic, 100 µM LY was passed through the apical channel at 120 μL/h, followed by sample collection from both the inlet and outlet reservoirs of the apical and basolateral channels. Prior to adding microvascular endothelial cells, samples were collected from chips containing only differentiated lung epithelial cells in the apical channel (monoculture, Day 19) and after the addition of microvascular endothelial cells (coculture, Day 21). Most chips were observed to have a low P_app_; see Figure 2E of LY suggesting an intact barrier. Previous studies used a lung epithelial cell line (Calu-3 cells) in an insert platform set <2% LY transport as the threshold for an intact barrier of cells (Cingolani et al., 2019; Mukherjee et al., 2017). Therefore, chips with a % transport above this 2% threshold (Figure 2E, dotted line) were excluded from the drug permeability study. No significant difference was observed between the monoculture and the coculture MPS.

### Evaluation of drug absorbance to the MPS

**FIGURE 3:**
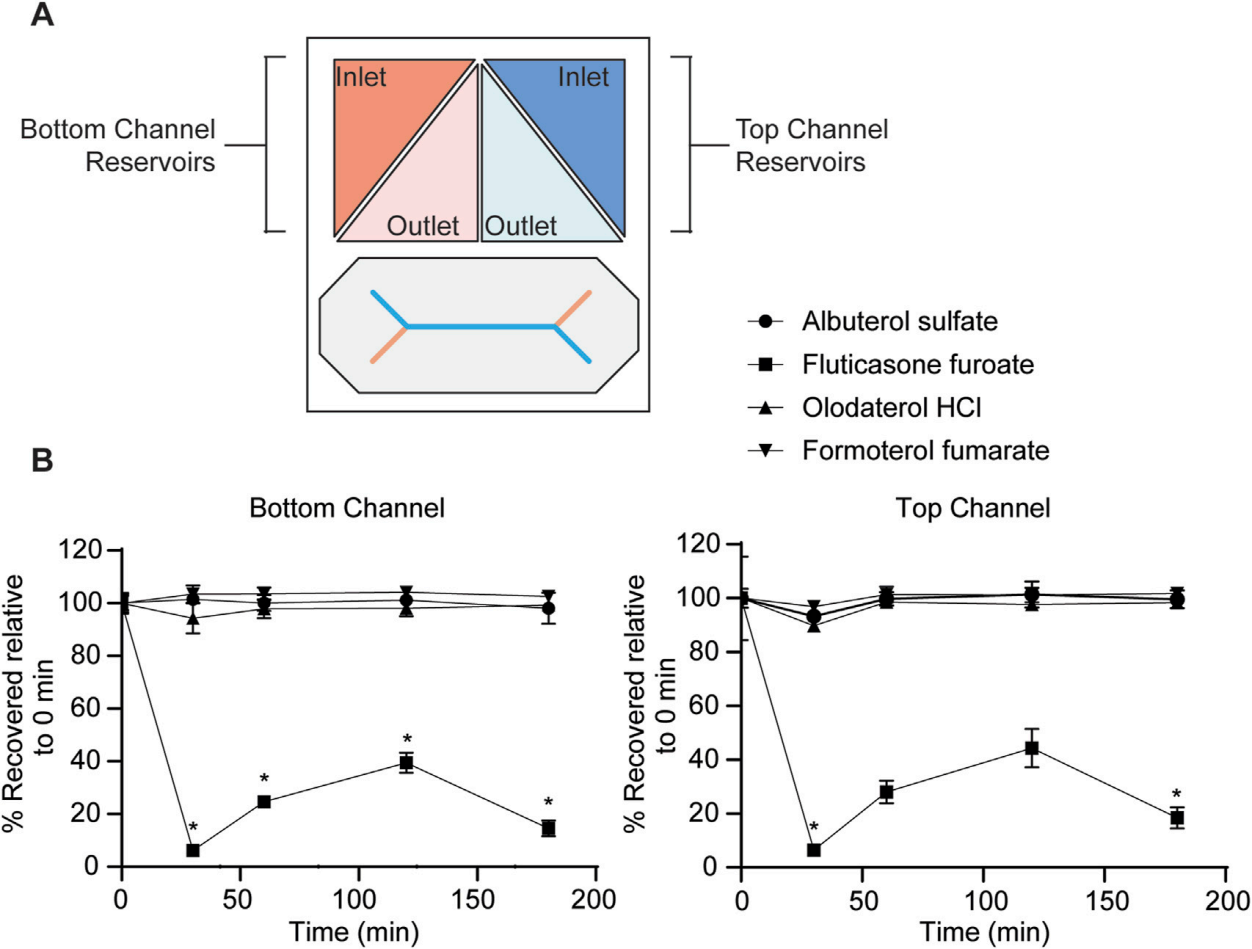
Evaluation of drug absorbance to the MPS. Samples were collected at 0, 30, 60, 120 and 180 min timepoints at a flow rate of 250 μL/h from the inlet and outlet reservoir shown in **(A)** from the apical and basolateral channel and quantitated with LC-MS/MS. **(B)** At each timepoint, drug concentrations (10 μM) were normalized to their respective starting concentration at time 0 min. Data represent the mean ± SD; n = 3 biological chip replicates; 2-way ANOVA, Time 0 vs. Time 30, 60, 120, 180 min was compared using Dunnett’s multiple comparison test, *p-value <0.05.

Next, drug absorbance to the MPS was evaluated. The small airway MPS is composed of PDMS, a hydrophobic material known to bind many lipophilic drugs, thus reducing the amount of free drug available to the cells (Leung et al., 2022; van Meer et al., 2017; van Midwoud et al., 2012). Given the range of solubilities and physiochemical characteristics, some lipophilic drugs with a higher log P may bind to the system (Table 1). To identify the amount of drug loss due to binding, we passed the drugs in solution through the MPS without cells and measured the amount recovered with LC-MS/MS. Each drug at 10 μM was flowed through both the top and bottom channels and samples were collected from the top and bottom, inlet and outlet reservoir at 0, 30, 60, 120 and 180 min timepoints (Figure 3A). Albuterol sulfate, olodaterol HCl, and formoterol fumarate was observed to have very little binding as there was no significant difference observed between the drug concentration recovered at each timepoint and time 0 min (Figure 3B). However, only 6%–44% of fluticasone furoate was recovered at 30 and 120 min respectively suggesting that the drug was significantly absorbed to the MPS. Given this data, permeability experiments with fluticasone furoate were not performed as the results would not provide an accurate measure of P_app_ due to the nonspecific binding.

**TABLE 1:** Drugs used in permeability study and their corresponding log P (Knox et al., 2024).

Drug	MW (g/mol)	Log P	Vendor	Cat #	Lot #
Albuterol sulfate	337.4	0.44	Selleckchem	S2507	S250704
Fluticasone furoate	538.6	3.73	Selleckchem	S6487	S648701
Olodaterol HCl	422.9	1.84	Selleckchem	S5925	S592501
Formoterol fumarate	840.9	1.91	USP	1286107	R097M0

### Evaluation of drug permeability

**FIGURE 4:**
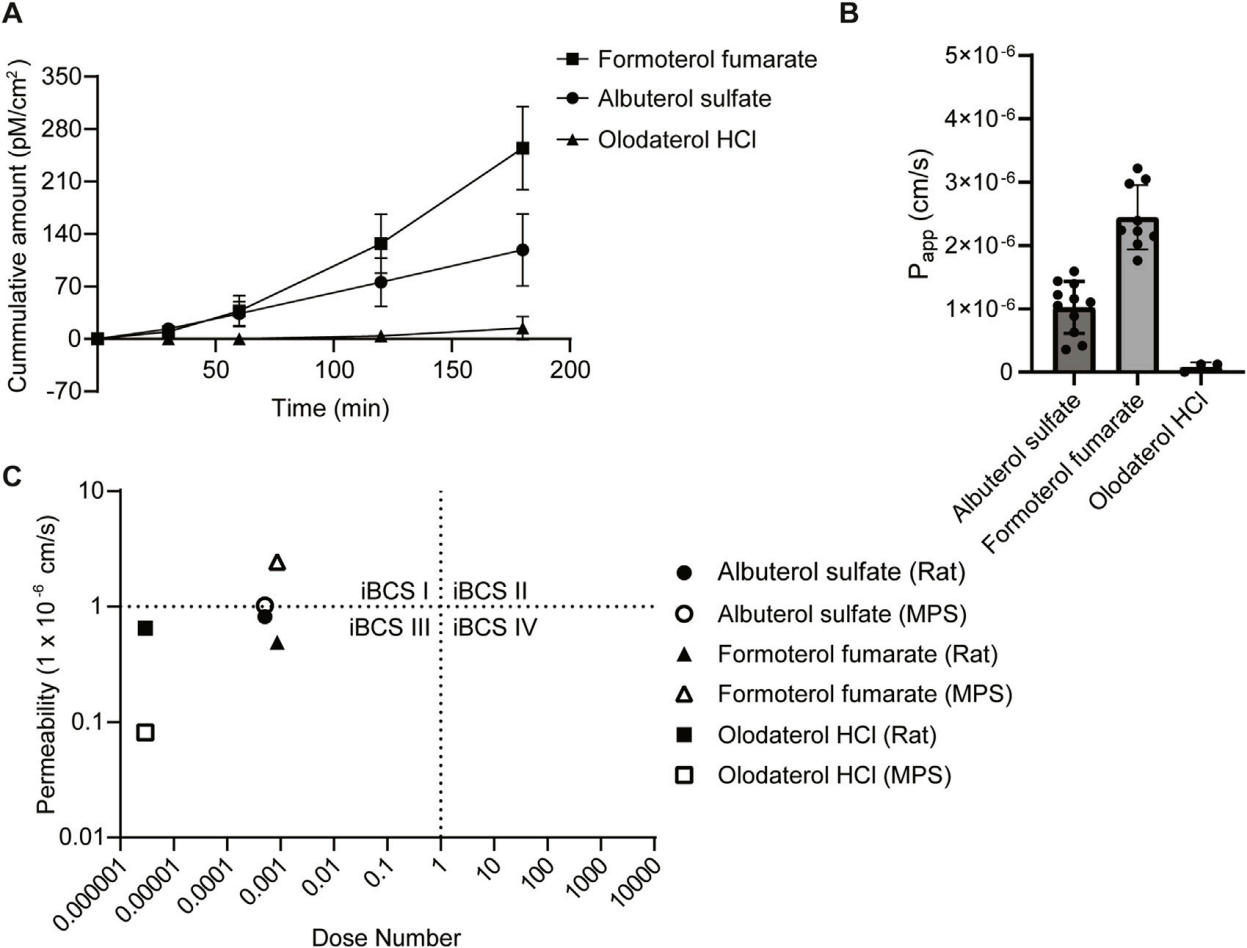
Assessment of drug permeability. **(A)** Samples were collected from the apical and basolateral inlet and outlet reservoir at 0, 30, 60, 120 and 180 min timepoints and quantitated with LC-MS/MS, Data represent the mean ± SD; (n = 3–11 chips). **(B)** The apparent permeability (P_app_) was calculated based on the concentration of drug recovered from the outlet of the apical and basolateral channels (See Equation 1 in methods). Chips that had a concentration below the limit of detection (<10 nM) were set to 0, Data represent the mean ± SD; (n = 3–11 chips). **(C)** Using the P_app_ and the dose number calculated using data from Table 2 and Equation 3, each drug of interest was categorized using the inhaled biopharmaceutical classification system (iBSC). In addition, permeability data from the literature using an *ex vivo* rat isolated perfused lung model was also categorized for comparison.

Finally, the permeability of albuterol sulfate, formoterol fumarate, and olodaterol HCL using the small airway MPS was evaluated. We then attempted to categorize our results based on an approach similar to what was done using a proposed inhaled biopharmaceutical classification system (iBSCs) (Bäckman et al., 2022; Hastedt et al., 2022; 2024). In addition, we compared our apparent permeability values to the effective permeabilities from an *ex vivo* IPL rat model from another study (Eriksson et al., 2018). A drug concentration of 10 µM was identified based on estimates of regional lung surface area, mucus thickness, mucus volume, and drug deposition as well as other *in vitro* permeability studies (Bäckman et al., 2022; Eriksson et al., 2018; Salomon et al., 2015). Small airway MPS containing both matured lung epithelial and microvascular endothelial cells with low LY transport (below 2%) were used to examine drug permeability. The cumulative amount of drug transported was 118 pM/cm^2^ (albuterol sulfate), 254 pM/cm^2^ (formoterol fumarate), and 14.4 pM/cm^2^ (olodaterol HCl) (Figure 4A). Using this information, we calculated an apparent permeability (P_app_) of 1.02 × 10^−6^ cm/s for albuterol sulfate, 0.0813 × 10^−6^ cm/s for olodaterol HCl, and 2.44 × 10^−6^ cm/s for formoterol fumarate (Figure 4B). To help to contextualize these permeability measurements, each drug was assigned a class using the recently proposed iBCS (Figure 4C) (Bäckman et al., 2022; Hastedt et al., 2022; 2024). Based on the apparent permeability measured in this study, rather than the effective permeability, and the dose number calculated using the information in Table 2 and Equation 3, albuterol sulfate and formoterol fumarate were categorized in class I and olodaterol HCl in class II.

**TABLE 2:** Values used to calculate the dose number for each drug and apparent permeabilities evaluated with the small airway MPS or effective permeabilities from an *ex vivo* rat IPL model from another study (Eriksson et al., 2018).

Drug	Dose (μg)	Solubility (μg/mL)	Dose number (D_o_)	Apparent Permeability (P_app_) (x10^-6^ cm/s) (MPS)	Effective Permeability (P_eff_) (x10^-6^ cm/s) (Rat)
Albuterol sulfate	90	17,700	0.000508	1.02	0.82
Fluticasone furoate	100	0.02	500	N/A	3.5
Olodaterol HCl	2.5	85,000	0.00000294	0.0813	0.65
Formoterol fumarate	10	1,160	0.000862	2.44	0.49

## Discussion

We present data showing that the small airway MPS may be used to assess the permeability of inhaled drugs and identified quality control parameters in-line with the literature that may be used to pre-screen chips. However, the nonspecific binding of lipophilic drugs like fluticasone furoate to PDMS presented a challenge for estimating their permeability, and reliable results were obtained only with albuterol sulfate, formoterol fumarate, and olodaterol HCl.

### Characterization of the small airway MPS

The small airway MPS was characterized using criteria from established *in vitro* models to enhance the robustness of drug permeability studies. This includes observing the presence of relevant cell types observed *in vivo* or in other *in vitro* models and demonstrating an intact barrier prior to drug permeability studies. In the small airway MPS, we show evidence of similar cell types generated in the lung, including goblet, basal, and ciliated cells (Figure 2A), consistent with published work (Benam et al., 2016). Mucin is also observed at a concentration of 1.12 mg/mL. This is much higher than a previous report from an airway chip using bronchial cells (Plebani et al., 2021). The discrepancy may be due to the amount of time the cells were maintained at ALI. For example, our chips were maintained for 14 days at ALI while others for 7 days before sampling for mucin. In addition, in our chips, mucus was allowed to accumulate for 3 days before washing.

RT-qPCR analysis showed expected differentiation-related changes in functional and cell type-specific gene markers, including MIK67 (cell proliferation) and EpCam (epithelial cells). We also show evidence of CTS markers associated with lung epithelial cells *in vivo*, including goblet, basal and ciliated cells, consistent with previous published work (Benam et al., 2016). Interestingly, some CTS genes and gene sets showed reduced expression after addition of endothelial cells (*i.e*., Day 22 compared to Day 18), especially in genes related to basal and ciliated cells. Whether this is due to MPS manipulations and/or a potential paracrine effect following addition of endothelial cells remains to be determined by future work.

Of the *SLC22* family genes encoding OCTs, *SLC22A1*, and *SLC22A3-A5* were detected using our screening assay in the small airway MPS, while *SLC22A2* (OCT2), and genes encoding organic anionic transporters *SLC22A6-9* (OATs)*,* were not detected, consistent with available RNA sequencing data from human lung tissue (Uhlén et al., 2015) and with the literature (Bosquillon, 2010; Gumbleton et al., 2011). While expression results infer *SLC22A1* (OCT1) and *SLC22A3* (OCT3) may be rare transcripts in small airway lung epithelia, tissue-specific expression (and its translation efficiency in that tissue) should be considered. For example, *SLC22A3* (OCT3) appears to be highly expressed (range 40–70 nTPMs) in alveolar type 2 epithelial cells (Uhlén et al., 2015), which may, in part, explain its lower detection in our assay. Thus, cell specificity as well as how a quantitative change in gene expression impacts protein level and function may also be important considerations. To our knowledge, this is the first study to evaluate transporter gene expression longitudinally in an *in vitro* small airway MPS. The screening assay used here was a candidate approach and sensitive for the detection of 36/51 candidate transporters (or 67/84 total gene targets) with good reproducibility, particularly for the most abundant transcripts (Supplementary Section S3B). For *SLC22A1* (OCT1) relative expression, which bordered the amplification cut-off for Days 4 and 22, we performed two additional independent experiments to confirm expression, as well as other transporters of interest (Supplementary Table ST4). While the present array was used as a screening tool to determine relative expression levels of candidate transporters, future work may consider methods of absolute qPCR quantification to provide more quantitative information about transporter expression in different MPS models.

To ensure consistent results regarding drug permeability, it is important to ensure an intact cell barrier between the apical and basolateral channels (Cingolani et al., 2019; Frost et al., 2019; Mukherjee et al., 2017). LY has been used as a quality control to assess barrier integrity in other *in vitro* models, such as assays using membrane inserts with either Caco-2 cells or Calu-3 cells (Cingolani et al., 2019; Fredlund et al., 2017). Less than 2% LY transport has been considered acceptable when using Calu-3 cells in a membrane insert model (Cingolani et al., 2019; Mukherjee et al., 2017). We found that most of our chips fell below that threshold suggesting an intact barrier (Figure 2E). Taken together, we show that our protocol incorporates both primary small airway epithelial and microvascular endothelial cells to generate the small airway MPS that meets our quality control criteria of observing similar cell types as other *in vitro* models.

### Study considerations

Before permeability studies were performed, drug stability was evaluated to identify any drug degradation in the Pneumacult basal media. Initial benchtop studies indicated that olodaterol HCL and formoterol fumarate were light sensitive. To address this concern, BSA was added to the samples prior to analytical evaluation. Future studies could also consider evaluating drug stability in their respective media. Next, the nonspecific binding of the four drugs was considered in the MPS composed of hydrophobic material PDMS. Lipophilic compounds often bind to hydrophobic materials like PDMS, which can affect the accuracy of many endpoint assays (Leung et al., 2022). Therefore, to evaluate the risk of a similar observation used in MPS, the drug absorbance was examined in the system to determine if the compounds were binding to the device. As expected, given the higher Log P of 3.73 (Table 1), significant binding of fluticasone furoate was observed to the MPS but insignificant amounts from the other more hydrophilic drugs (Figure 3B). The binding is most severe at the 30 min timepoint and decreases at 120 min. This is likely due to the drug binding sites becoming saturated as the drug continuously passes through the channel. Others have also observed severe drug binding on PDMS-based MPS and have demonstrated how to calculate for this loss (Carius et al., 2024; Shirure and George, 2017). However, given the severity of drug binding to the chip, the unbound drug concentration would not be physiologically relevant. Future drug permeability studies could consider using oxidative treatments to PDMS to reduce non-specific binding or chips composed of either glass or thermoplastics to help minimize drug binding (Campbell et al., 2021; Hirama et al., 2019; van Meer et al., 2017; van Midwoud et al., 2012). Chips constructed with an alternative material to PDMS that have less absorptive properties will be considered in subsequent studies. Taken together, these studies demonstrate the importance of developing quality controls to ensure accurate and consistent results.

While culturing the chip with primary human lung epithelial cells, a few challenges were encountered. For example, cell migration from the apical to the basolateral channel was often observed. This could be due to the migratory nature of the epithelial cells as well as the pore size (7 µm) between the channels (Nawroth et al., 2023). Others have encountered this difficulty as well and have suggested that migration can be minimized by coating the basolateral channel with the surfactant Pluronic^®^ (Nawroth et al., 2023). To mitigate this issue, the amount of ECM that got to the bottom channel was minimized by maintaining the bottom channel in air when coating the top channel with ECM. Additionally, the current study only included results from two lung epithelial cell donors. Attempts to culture other donor cells from different vendors were unsuccessful. While this may be a current limitation of the system, others have developed ways to amplify their donors of interest through inhibiting SMAD signaling, while still maintaining their differentiation potential (Levardon et al., 2018; Mou et al., 2016). This method could also be leveraged to amplify donors with a specific mutation to study genetic disorders like cystic fibrosis using the small airway MPS.

### Classification of inhaled drugs

Recently, an iBCS has been proposed to better understand the developmental, clinical, and regulatory risks of active pharmaceutical ingredients (APIs) in the lung (Bäckman et al., 2022; Hastedt et al., 2022; 2024). Similar to the original BCS system for orally ingested drugs in the gut (Amidon et al., 1995), the iBCS classifies inhaled drug products or APIs based on their permeability, solubility, and dissolution. A drug with an effective permeability above 1 × 10^−6^ cm/s indicates that the drug is completely absorbed and has a short luminal half-life, while below this value indicates that only a fraction is absorbed (<85%) and has a longer luminal half-life. Furthermore, a drug with a dose number (D_o_) above one indicates that only a fraction of the deposited drug can dissolve in the epithelial lining fluid (ELF), while a D_o_ below one indicates that the deposited drug can fully dissolve in the ELF (Hastedt et al., 2024). This new system accounts for differences between inhaled and orally ingested drugs. For example, most approved inhaled drugs act locally in the lung, unlike orally ingested drugs that target the systemic circulation (Hastedt et al., 2022). However, to classify inhaled drugs using the iBCS, the permeability or the rate at which a drug passes through a layer of cells needs to be evaluated.

Applying this framework, we categorized our drugs of interest using the P_app_ generated from the small airway MPS and the dose number calculated using data in Table 2 and Equation 3. The dose number accounts for the solubility, the mass of the drug deposited in a region of the lung (50% of the nominal dose) and the volume of available fluid for the drug to dissolve in the lung (Bäckman et al., 2022; Hastedt et al., 2024). Doses were obtained from drug labels (FDA.gov., 2014; FDA.gov., 2019b; FDA.gov., 2023; FDA.gov., 2019a). Based on our data, albuterol sulfate (1.02 × 10^−6^ cm/s) is categorized as class I but near the border of class I/III, olodaterol HCl (0.0813 × 10^−6^ cm/s) is in class II, and formoterol fumarate (2.44 × 10^−6^ cm/s) is in class I. In order to compare our results, the effective permeabilities from an *ex vivo* IPL rat model were also categorized (Figure 4C). These results differ slightly from the MPS: albuterol sulfate (0.82 × 10^−6^ cm/s) is still placed near the border of class I/III, and olodaterol HCl (0.65 × 10^−6^ cm/s) is still in class II; however, formoterol fumarate (0.49 × 10^−6^ cm/s) is categorized in class II instead of class I. In comparison of the two models, there are several differences that should be considered. For example, species-specific differences exist between rat and human cells. In addition, there are differences in the experimental setup, for example, the *ex vivo* rat model contained epithelial lining fluid (ELF), however in the small airway model, the ELF would have been washed away when treating the cells under a liquid-liquid interface. Furthermore, the small airway MPS was used to calculate an apparent permeability while the *ex vivo* rat model is used to calculate an effective permeability. Future work may consider a more careful comparison between these two models as suggesting that the small airway MPS is superior or inferior to an *ex vivo* rat IPL model is beyond the scope of this study.

While there were minimal differences between permeabilities measured with the *ex vivo* IPL rat model and the small airway MPS, it may still be advantageous to consider the small airway MPS with human primary cells for evaluating the permeability of inhaled drugs. MPS provides a bridge between preclinical and clinical development by providing more physiologically relevant data compared to traditional *in vitro* models. Permeability data from MPS help estimate local vs systemic exposure for inhaled drugs which informs dosing strategies in early phase clinical trials. Additionally, apparent permeability measurements from the MPS may serve as useful inputs to physiologically based pharmacokinetic (PBPK) models for orally inhaled drug products. These models may be used to support product development, or to answer regulatory questions such as the relationship between pharmacokinetics (PK) study metrics and regional lung drug delivery or the need for generic orally inhaled drug product developers to conduct PK studies with a charcoal block to quantify total lung exposure. Moreover, leveraging MPS with primary human cells may be advantageous as the PBPK model Mimetikos Preludium™ (Emmace Consulting AB, Lund, Sweden) used to create the boundaries between the four classes relies on human physiological characteristics like lung surface area and epithelial lining fluid (ELF) volume (Bäckman et al., 2022). In addition, MPS supports reducing, replacing, and refining animal models in drug development, and can be seeded with human cells from a specific region of the lung enabling more precise permeability measurements. MPS have the potential to reduce reliance on animal studies used in the development of direct pulmonary delivery, however additional studies should be considered.

This study, while limited in the number of drugs evaluated, provides a basic paradigm to culture, characterize, and evaluate drug permeability with the small airway MPS. Future work could consider modifications to the small airway MPS that address limitations identified in this study and a broader evaluation of the system using a larger range of inhaled drugs.

## Data Availability

The original contributions presented in the study are publicly available. This data can be found here: https://doi.org/10.6084/m9.figshare.29437163.v2
