# Supplementary material for: Assessment of drug permeability using a small airway microphysiological system
Source: Front Pharmacol. 2025 Jul 17;16:1621775. doi: 10.3389/fphar.2025.1621775 (PMC12312011; doi:10.3389/fphar.2025.1621775)
Supplement: Supplementary file 1 [file DataSheet2.docx]

**Supplementary Material**

**Assessment of Drug Permeability using a Small-Airway Microphysiological system**

**Supplementary Methods & Results Sections (S)**

**S1. RNA Extraction and Data Analysis**

Epithelial cells were washed 2x with cold PBS (Gibco, cat # 10010023) after which, 100 µl of TRIzol (Invitrogen cat# 15596018) was added to the upper channel and incubated at RT for 10 min with intermittent pipetting to lyse epithelial cells from Day 4 and Day 18 chips (which do contain endothelial cells). On Day 22 (which contains endothelial cells) TRIzol was added to the epithelial cell channel before or after lysis of endothelial cells (data not shown here). Intermittent pipetting was used to help cell lysis. After collecting the epithelial cell lysate, the channel was washed with an additional 100 µl of TRIzol which was combined with the previous lysate. To collect epithelial cells that may have detached during the PBS wash, cell suspensions were pelleted at 400 x g for 5 min at 4°C. After removing the PBS, the pellet was resuspended and incubated in 300 µl TRIzol at RT for 5 min and then combined with the 200 µl of Trizol-lysate previously prepared from the respective chip (making a total volume of 500 µl lysate per chip). Then 100 µl of chloroform (Sigma cat# C2432) was added to the lysate and mixed by inversion (15x) before incubating the mixture at RT for 3 min. The mixture was then centrifuged at 12,000 x g for 15 minutes at 4°C (phase separation) before transferring the upper aqueous phase of each sample to a PureLink spin column (Thermo, cat# 12183018A) and purifying the RNA according to the kit protocol followed by elution of RNA in 30 µl of nuclease-free water. RNA concentration, purity, and contaminants were determined via the Thermo Fisher NanoDrop 8000 spectrophotometer and RNA integrity was assessed via Agilent 2100 bioanalyzer (Agilent RNA Nano Kit, cat # 5067-1511). For all samples, RNAs were diluted to the same concentration, aliquoted, and stored at -80°C to avoid freeze-thaw cycle effects and variability between cDNA synthesis reactions.

Prior to cDNA synthesis, total genomic DNA (gDNA) was eliminated from all RNA samples using QuantiNova reverse transcription kit (Qiagen, cat# 205411) according to manufacturer’s instructions. For cDNA synthesis, 400 ng of RNA was used as the template and the QuantiNova reverse transcription kit was used as per manufacturer’s instructions. SYBR green qPCR reactions were prepared according to the manufacturer’s instructions (QuantiNova SYBR Green qPCR Master Mix (Qiagen, cat# 208056)). PCR cycling conditions specified for QuantiNova LNA PCR arrays were used and threshold cycles (Cts) were defined by applying a threshold intersection value of 0.06 to the amplification plot. Melt curves were manually examined to ensure the presence of a single amplicon. Three candidate gene targets (*ABBC10, ABCD1, and ABCA4*) were excluded from data analysis due the presence of primer dimer artifacts (**See Section S3C**).

Qiagen GeneGlobe (<https://geneglobe.qiagen.com/re>) was used for data analysis using a default Ct cut-off of 35. Cts observed above the cut-off were set to 35 for analysis as per GeneGlobe’s recommendation. Targets with Cts > 35 in at least 95% of RT-qPCR samples across all experimental conditions were not used in differential gene expression analysis (**See Section S3C**). Expression data was normalized to the collective average of *β-actin* (*ACTB*), *Glyceraldehyde-3 phosphate dehydrogenase* (*GAPDH*), and β-*2-microglobulin* (*B2M*) genes which were found to be the most stably expressed set of housekeeping genes (HKGs) across the experimental conditions (**See section S3A**). The difference between ΔCT values for Days 18 and 22 vs Day 4, respectively, was represented by ΔΔCT values, where fold-change was calculated using 2^-ΔΔCT^ (Livak & Schmittgen, 2001; Schmittgen & Livak, 2008). Log2 fold changes are shown in **Figure 2** and raw fold changes are provided in **Supplementary Tables ST2 and ST3**. Only samples that passed the QC threshold for gDNA contamination (Ct > 33), PCR amplification, and RT efficiency (as determined by GeneGlobe) were included for further analyses. One technical replicate from experiment 1 (Day 4) was excluded due to detectable levels of gDNA.

**S2. Biological and Technical Replicates and qPCR Array Design**

Three independent chips were collected on Days 4, 18, and 22. RNA from each chip was converted into 3 independent cDNA replicas and each cDNA was evaluated by qPCR on a separate/independent array (*i.e*., 3 technical cDNA replicates per chip/RNA). Per experiment, each technical replicate was performed on different days as described in detail in **Figure S2**. cDNAs were immediately frozen following synthesis and thawed once before using to prepare SYBR green qPCR reactions. SYBR green qPCR reactions were prepared for each cDNA within a technical replicate using the same master mix and immediately analyzed on the designated qPCR array (**see Figure S2**). cDNAs generated from a commercial control RNA (Thermo Fisher cat# QS0639) on the same day as the test RNAs served as an inter-array control on each qPCR array to assess array-to-array variability.


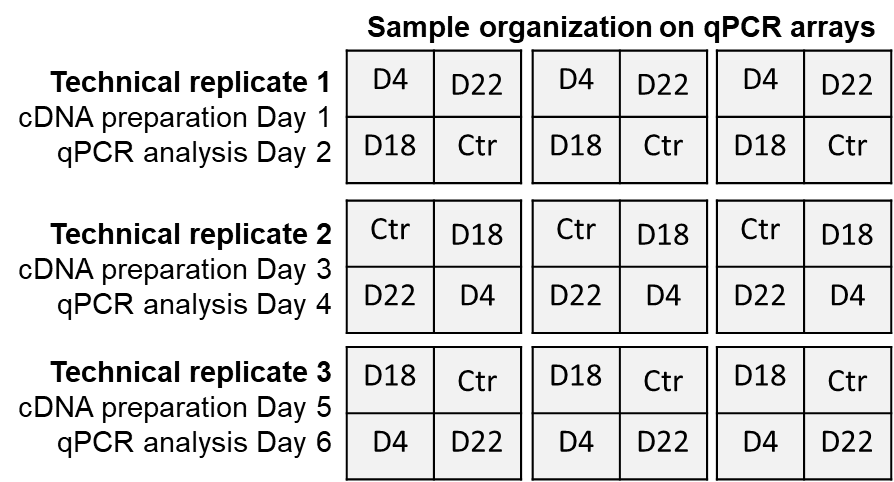


**Figure S2.** **Schematic representation of experimental design illustrating organization of biological and technical replicates on custom qPCR arrays.** For each technical replicate, three cDNAs were generated from each RNA sample from each of the 3 independent chips (per experimental condition) and were included on separate arrays that were run on the same day to reduce systematic error or batch effect(s). D4 = Day 4, D18 = Day 18, D22 = Day 22, Ctr = commercial control RNA.

***S3. Quality Control (QC) Assessment of qPCR Arrays***

*A. Data normalization and transformation of housekeeping (HKGs)*

The ideal reference gene for normalization should exhibit stable expression across samples, irrespective of cell type or experimental conditions (Rydbirk et al., 2016). However, relying on a single HKG for normalization can lead to errors due to variability in gene expression across different cell/tissue sources and experimental conditions. Thus, using more than one HKG or a normalization factor representing the average of multiple stably expressed HKGs can provide more reliable and accurate normalization in qRT- PCR (Vandesompele et al., 2002). To assess and validate the HKGs on our custom QuantiNova LNA qPCR array (Qiagen, LLC), we analyzed the Ct values to assess stable expression across multiple days and samples, as completed in a previous study (Aithal & Rajeswari, 2015). In this study, the average expression of the *ACTB, GAPDH,* and *B2M* genes was identified as the most suitable HKG factor for data normalization, as these targets exhibited the most stable expression across all experimental conditions (Figure S3A, left panel). This HKG factor also showed minimal fold change differences in expression between Day 18 (1.01) and Day 22 (1.08), relative to Day 4 (baseline), respectively (Figure S3A, right panel).

**
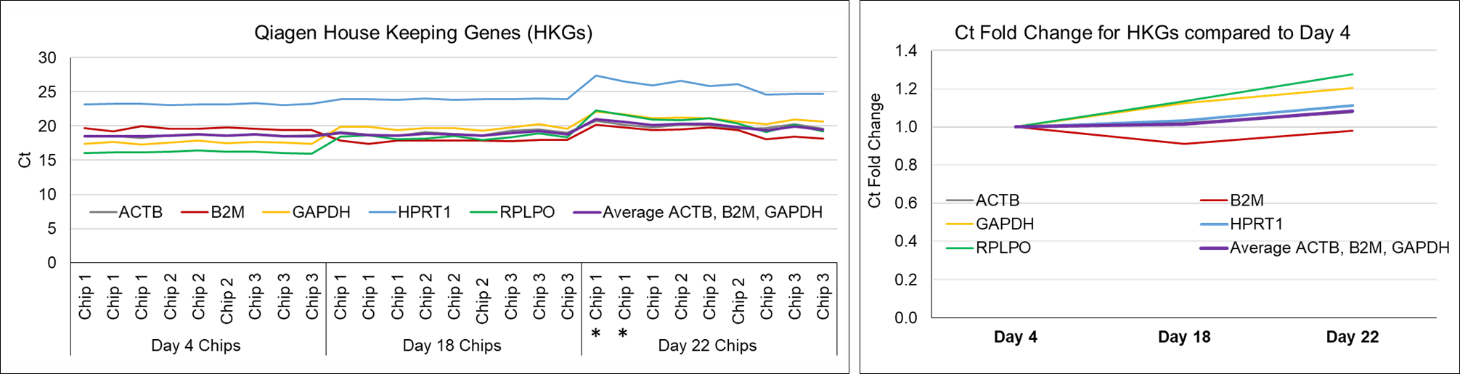
**

**Figure S3A. Identification of suitable HKGs or a HKG factor for data normalization**. **Left panel**: Ct values are shown for all HKGs (including the HKG factor or average of *ACTB, GAPDH*, and *B2M*) across all experimental samples and biological/technical replicates. Of note, on Day 22, two technical replicates of Chip 1* were identified as outliers following PCA analysis (see Section S3D). A sensitivity analysis with and without these samples did not significantly impact the stable expression of individual HKGs or the selected HKG factor. **Right panel**: Illustrates the average Ct fold change values for all HKGs and the HKG factor (i.e., *ACTB, GAPDH*, and *B2M*) between Day 18 and Day 22 chips relative to Day 4 chips (baseline samples), respectively.

*B. Precision and reproducibility*

To assess data reproducibility (*e.g.,* inter-assay variance (Bustin et al., 2009)), we determined the distribution of standard deviation for Δ Cts for a universal control RNA and for experimental samples. This data provides information on the variability of cDNA synthesis reactions, array-to-array variability and, for experimental samples, biological variability. For experiment 1, a total of 9 arrays were analyzed over 3 days (3 arrays per day) with each one containing a control cDNA generated from a commercial RNA sample (**see Section S2 for array design and S3C for description of RNA sample**).


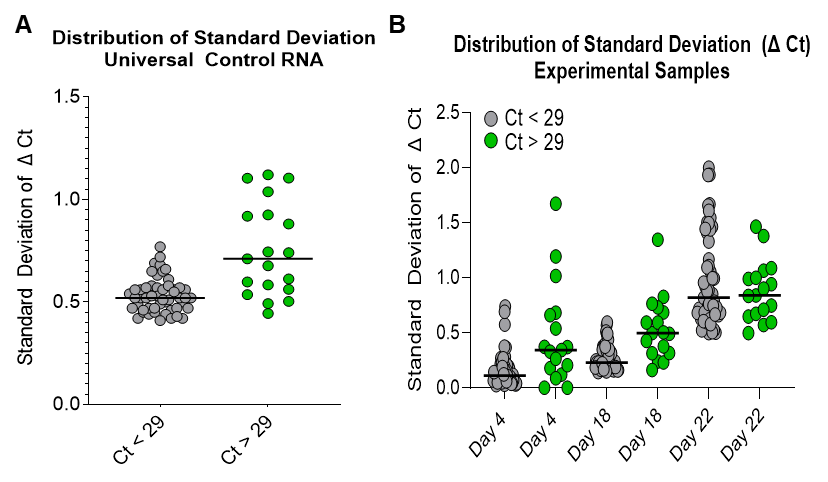


**Figure S3B. The distribution of standard deviation for Δ Cts is shown for the control RNA (A) and experimental test samples (B).** Targets are divided based on Ct values (> or < 29). Note that targets with higher Ct values, which are less abundant, tend to have a wider distribution of standard deviation and are more variable.

C. Primer efficiency and Specificity

Because the QuantiNova LNA qPCR assays (Qiagen LLC) were developed using stringent design criteria and laboratory-validated algorithms, with predicted primer efficiencies within the ideal range of 90% to 110%, even for targets with high GC content (data shown at [Qiagen URL](https://www.qiagen.com/us/products/discovery-and-translational-research/pcr-qpcr-dpcr/qpcr-assays-and-instruments/mrna-incrna-qpcr-assays-panels/quantinova-lna-pcr-assay)) - we did not assess primer efficiencies internally. The incorporation of Locked Nucleic Acid (LNA) bases further enhances primer specificity and sensitivity, enabling accurate detection across a wide dynamic range. For detailed information on assay design and performance characteristics, please refer to the product documentation at the [Qiagen website](https://www.qiagen.com/us/products/discovery-and-translational-research/pcr-qpcr-dpcr/qpcr-assays-and-instruments/mrna-incrna-qpcr-assays-panels/quantinova-lna-pcr-assay).

On the custom array (Qiagen cat# 249970 SBCA02438), most primer assays (83/89 including HKGs) targeted regions spanning intron-exon boundaries (**Supplemental Table ST1**) with good coverage of biologically relevant transcripts for the target gene of interest. Though the risk of gDNA amplification was higher in assays targeting sequences within exons (n= 6), a gDNA removal step was completed for all samples prior to cDNA synthesis and a control assay for detection of gDNA was also included on the qPCR array. Only samples that passed QC for gDNA contamination were included in analyses. We experimentally assessed primer specificity by running a “no template control” (NTC, where water was used in place of RNA as the template for cDNA synthesis reactions). A total of 6 NTCs were analyzed on the qPCR arrays with only three genes/targets showing consistent signal below the Ct cut-off (35) which was due to primer dimers (**see Figure S3C**). These genes were excluded from further analysis.

**
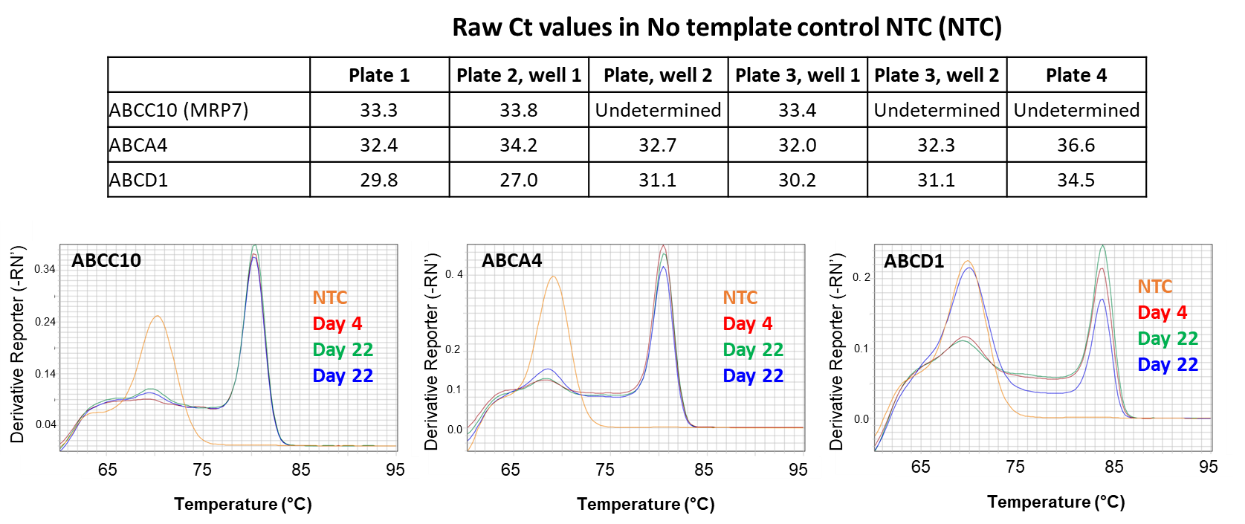
**

**Figure S3C. Nonspecific signal from primer dimers for 3 sets of primers**. The table shows gene targets for which primers yielded Ct values below the Ct cut-off (35) in NTC samples (Ct values are provided). Melt curves for these targets show a single peak in the NTC and two peaks in experimental samples, characteristic of primer dimers with the peak at the lower temperature corresponding to the smaller primer dimer (Ririe et al., 1997).

To evaluate the assay’s ability to identify true negatives, we compared our results with an online publicly available database that provides transcript abundance based on mRNA-seq studies of human lung tissue (<https://www.proteinatlas.org/>). Table S3C (below) lists targets that were below the limit of detection (Ct “undetermined” or ≥ 35) in our assay in at least 95% of all experimental samples (n = 14). Detection of the target in the commercial control RNA (average Ct from 9 qPCR replicas) together with expression in the lung *in vivo* (normalized transcripts per million, nTPMs) are indicated. Most targets that were undetectable in our assay (9/14) were not detected in the lung *in vivo* while 4 genes were detected at very low read levels *in vivo*, 3 of which were efficiently detected in the control RNA (see Table S3C). Notably, the commercial RNA (Thermo Fisher cat# QS0639) used in this study as an experimental control included equal quantities of RNA pooled from 10 different cell types or cell lines: Adenocarcinoma (mammary gland); Melanoma; Hepatoblastoma (liver); Liposarcoma; Adenocarcinoma (cervix); Histiocytic lymphoma; Embryonal carcinoma (testis); Lymphoblastic leukemia (T lymphoblast); Glioblastoma (brain); Plasmacytoma.

Of note, one target, *SLCO2B1*, which was undetectable in our test samples under the conditions used here, showed moderate expression (58.8 nTPM *) in the lung *in vivo*, however, further analyses using available single cell-RNA-seq data (<https://www.proteinatlas.org/humanproteome/single+cell>) revealed that it is highly enriched in infiltrating macrophages with low detection (< 10 nTPM) in lung epithelial cells.

**Table S3C. Expression of targets below our limit of detection in the control RNA and the lung *in vivo*.**

| **Target** | **Reason for exclusion** | **Av Ct in Control RNA** | **Expression in whole lung tissue (nTPM)** |
| --- | --- | --- | --- |
| ABCB5 | Below limit of detection | 28 | 0.1 |
| ABCC11 | Below limit of detection | Below limit of detection | 0 |
| ABCC12 | Below limit of detection | Below limit of detection | 0 |
| ABCG8 | Below limit of detection | 29 | 0 |
| SLC22A2 | Below limit of detection | Below limit of detection | 0 |
| SLC22A6 | Below limit of detection | Below limit of detection | 0 |
| SLC22A7 | Below limit of detection | 29 | 0 |
| SLC22A8 | Below limit of detection | Below limit of detection | 0 |
| SLC22A9 | Below limit of detection | 29 | 0 |
| SLC10A2 | Below limit of detection | Below limit of detection | 0.1 |
| SLCO1A2 | Below limit of detection | 27 | 2.5 |
| SLCO2B1 | Below limit of detection | 31 | 58.8 * |
| TRPM5 | Below limit of detection | Below limit of detection | 0 |
| TUBB4A | Below limit of detection | 27 | 0.6 |

*D. Principal component analysis (PCA)*

PCA of expressed genes (n= 67) across the three experimental conditions was performed on data from 35 samples to identify batch effects and outliers. As previously noted in **Section S1**, one sample was excluded from further analyses due to gDNA contamination. PCA analysis showed clear separation of the commercial RNA control samples and three clusters for test samples - with Days 18 and 22 clustering closer together compared to Day 4. PCA further indicated two potential outlier samples for Day 22 (samples 19 and 20, **Figure S3D**), which in general showed more variability compared to samples from Day 4 and Day 18. However, a sensitivity analysis excluding these samples (19 and 20) did not alter results significanlty; in fact, top differentially expressed hits remained statistically significant. As a result, these samples were not excluded from analyses.


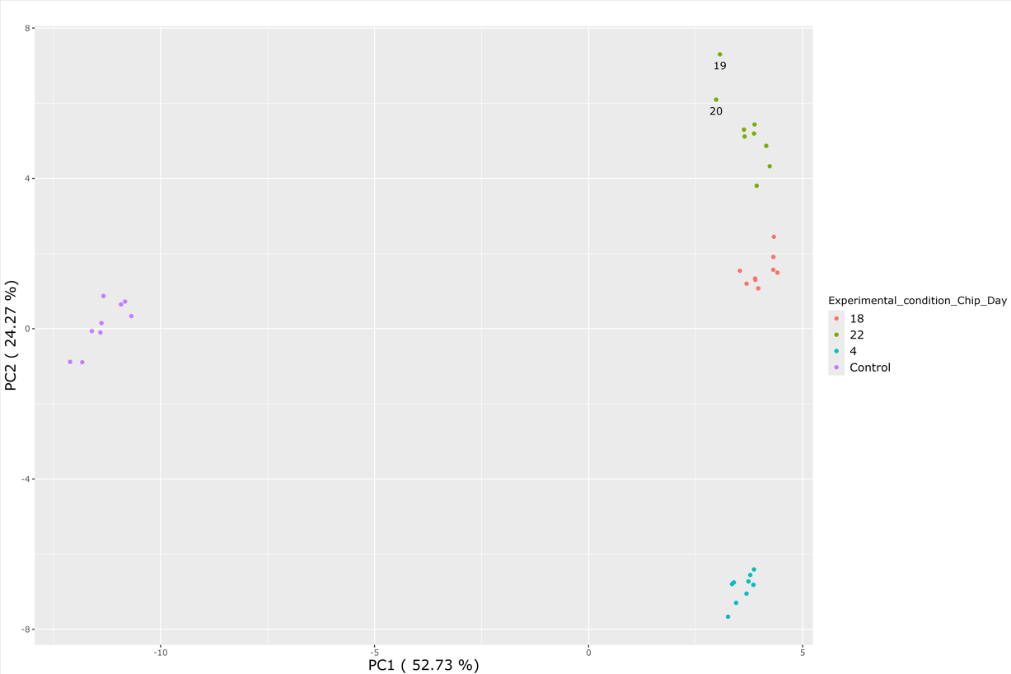


**Figure S3D.** **PCA of all samples based on Δ CTs of candidate targets expressed across all experimental conditions**. The two outlier samples from Day 22 are indicated.

**S4. Analysis of Baseline Samples Used for Differential Gene Expression**

Since Day 4 was used as the baseline or reference for differential gene expression, we examined the extent of variance in normalized, relative expression values (Δ Cts) across Day 4 samples using PCA (**see** **Section S3D**) and the spread of Δ Ct standard deviation (**see Section S3B**). **Figure S4** also shows consistent changes in expression for cell type specific (CTS), proliferative, and drug transporter (47/51) genes by magnitude and direction of expression for each sample/chip.

**
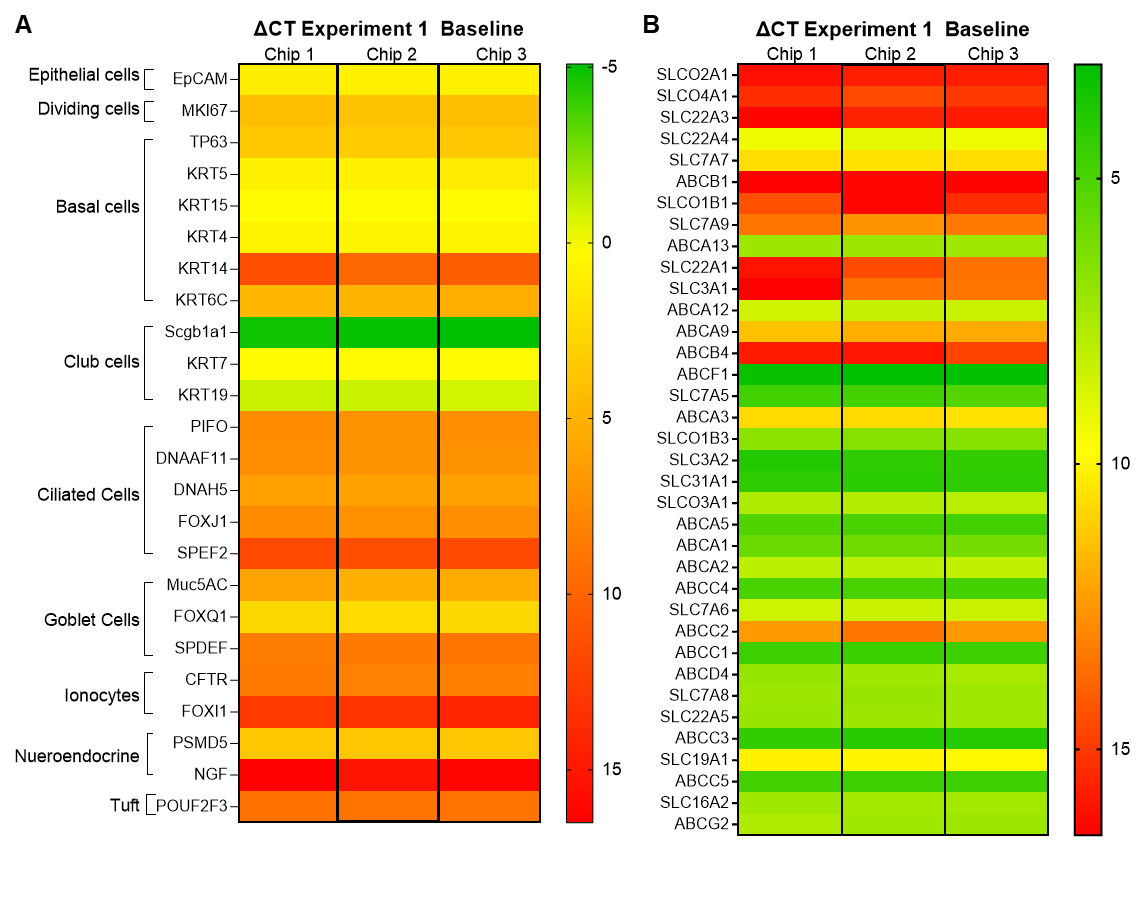
Figure S4.** **Day 4 sample variance**. Δ Ct values for select lung cell type and proliferation gene markers (A) and select drug transporters (B) from all Day 4 chips (average of technical replicates for each chip are shown). The most abundant transcripts are shown in green and the least abundant in red.

**References**

Aithal, M. G., & Rajeswari, N. (2015). Validation of housekeeping genes for gene expression analysis in glioblastoma using quantitative real-time polymerase chain reaction. *Brain Tumor Res Treat*, *3*(1), 24-29. <https://doi.org/10.14791/btrt.2015.3.1.24>

Bustin, S. A., Benes, V., Garson, J. A., Hellemans, J., Huggett, J., Kubista, M., Mueller, R., Nolan, T., Pfaffl, M. W., Shipley, G. L., Vandesompele, J., & Wittwer, C. T. (2009). The MIQE guidelines: minimum information for publication of quantitative real-time PCR experiments. *Clin Chem*, *55*(4), 611-622. <https://doi.org/10.1373/clinchem.2008.112797>

Livak, K. J., & Schmittgen, T. D. (2001). Analysis of relative gene expression data using real-time quantitative PCR and the 2(-Delta Delta C(T)) Method. *Methods*, *25*(4), 402-408. <https://doi.org/10.1006/meth.2001.1262>

Ririe, K. M., Rasmussen, R. P., & Wittwer, C. T. (1997). Product differentiation by analysis of DNA melting curves during the polymerase chain reaction. *Anal Biochem*, *245*(2), 154-160. <https://doi.org/10.1006/abio.1996.9916>

Rydbirk, R., Folke, J., Winge, K., Aznar, S., Pakkenberg, B., & Brudek, T. (2016). Assessment of brain reference genes for RT-qPCR studies in neurodegenerative diseases. *Sci Rep*, *6*, 37116. <https://doi.org/10.1038/srep37116>

Schmittgen, T. D., & Livak, K. J. (2008). Analyzing real-time PCR data by the comparative C(T) method. *Nat Protoc*, *3*(6), 1101-1108. <https://doi.org/10.1038/nprot.2008.73>

Vandesompele, J., De Preter, K., Pattyn, F., Poppe, B., Van Roy, N., De Paepe, A., & Speleman, F. (2002). Accurate normalization of real-time quantitative RT-PCR data by geometric averaging of multiple internal control genes. *Genome Biol*, *3*(7), RESEARCH0034. <https://doi.org/10.1186/gb-2002-3-7-research0034>
